# Importance of Maternal Persistence in Young Children's Persistence

**DOI:** 10.3389/fpsyg.2021.726583

**Published:** 2021-10-14

**Authors:** Masahiro Imafuku, Atsuko Saito, Kenchi Hosokawa, Kazuo Okanoya, Chihiro Hosoda

**Affiliations:** ^1^Department of Early Childhood Education and Care, Faculty of Education, Musashino University, Tokyo, Japan; ^2^Department of Psychology, Faculty of Human Sciences, Sophia University, Tokyo, Japan; ^3^Advanced Comprehensive Research Organization, Teikyo University, Tokyo, Japan; ^4^Department of Life Science Graduate School of Arts and Sciences, The University of Tokyo, Tokyo, Japan

**Keywords:** persistence, parenting styles, self-control, social development, preschooler

## Abstract

Persistence of a distant goal is an important personality trait that determines academic and social success. Recent studies have shown that individual differences in persistence involve both genetic and environmental factors; however, these studies have not examined the role of maternal factors on a young children's persistence. The present study examined whether mothers' persistence is associated with persistence in children aged 3–6 years. In addition, the associations between mothers' persistence/parenting style and children's self-control/social development (prosocial behaviors and difficulties) were examined. Our results showed that maternal persistence is essential for the child's persistence. Children's self-control and social development were also associated with the mothers' persistence and parenting style. Our findings suggest that a young child's persistence may develop under the influence of a familiar adult (i.e., mother) and characterizes their social development, highlighting the importance of persistence in parenting.

## Introduction

Persistence is the tendency to pursue long-term challenging goals. Persistence of a distant goal is not only a powerful measure of the classroom in children's engagement and a robust predictor of academic achievement but also a social success (Duckworth et al., 2007; Duckworth and Gross, 2014; Eskreis-Winkler et al., 2014; Li et al., 2018). However, the persistence of a goal differs substantially between individuals that may arise from both genes and environment (Rimfeld et al., 2016).

A few studies have shown that parents' nurturing attitudes are also significant for the children's persistence. Banerjee and Tamis-LeMonda (2007) found that strong persistence in children is developed between 6 and 14 months and that in low-income mother–infant dyads, infants with strong persistence in the exploration of their toys were raised by mothers who were sensitive and responsive to their infants' emotions and behaviors. In a novel study, adults' efforts promoted attempts to achieve the goal of children at 15 months (Leonard et al., 2017). It was also revealed that the amount of praise from caregivers for their efforts, such as “good job,” predicted 18-month-old children's persistence (Lucca et al., 2019).

Although these findings have suggested that caregivers serve as role models for children (Bandura, 1977, 1986), no studies have directly examined the relationship between parents' own persistence (not the persistence response for childcare) and children's persistence. Moreover, Lucca and Sommerville (2018) only argued that parenting has an impact on children's persistence, while parents' personality is associated with the development of adolescent personality traits (Schofield et al., 2012). Therefore, the persistence of the caregiver (particularly the mother who spends a lot of time with the child compared to father, at least in Japan) is likely to determine the degree of the child's persistence.

Previous studies have shown that supportive and responsive parenting reduces children's behavioral problems and promotes prosocial behavior and self-control (the ability to control one's behavior when exposed to temptations; Baumrind, 1971, 1991; Bornstein, 2006; Duckworth and Gross, 2014). At the same time, responsive and supportive parenting is a persevering task, especially in parenting young children. Therefore, parental persistence is likely to be associated with not only children's persistence but also their self-control and social development. In the meta-analysis, the results of the relationship between responsiveness and self-control in parenting varied from study to study. One study found that the association between responsiveness and self-regulation (e.g., compliance, inhibition, and emotion regulation) was not found among adolescents aged 10–22 years (Li et al., 2019), while another study found no association between responsiveness and self-regulation (e.g., compliance, inhibition, and emotion regulation) among preschoolers (Karreman et al., 2006). However, it remains unclear whether the persistence of parents is associated with both children's self-control and social development, such as behavioral difficulties and prosocial behaviors.

The present study aimed to clarify maternal persistence and parenting style as important environmental factors in the young children's persistence, self-control, behavioral difficulties, and prosocial behaviors. We addressed the following hypotheses: First, maternal persistence and parenting style were the key environmental factors in the child persistence. That is, the mother's persistence was associated with the child's (3–6 years old) persistence because beyond the mother's imitation, as the mother's persistent behavior of the goal and responsive teaching lead to promote the child's persistence (Banerjee and Tamis-LeMonda, 2007; Leonard et al., 2017). In the present study, persistence was measured using the short grit scale (Grit-S) (Duckworth and Quinn, 2009), which has been used to measure persistence in many studies in adults and children and has confirmed validity. Second, as the mother's parenting style was associated with the child's behavioral traits (Baumrind, 1991; Weiss and Schwarz, 1996; Alizadeh et al., 2011), parenting factors (mother's persistence and parenting style) also were associated with the child's self-control, behavioral difficulties, and prosocial behaviors. In the current study, we used an effortful control assessment to measure the temperamental aspect of self-control (Rothbart et al., 2001).

## Materials and Methods

### Participants

Through a web survey, a total of 107 Japanese mothers who lived with at least one child participated in our study (mean age = 35.0, range = 25.2–45.0 years, *SD* = 4.7). They were recruited by a company specializing in surveys (100-person surveys). If the mother has more than one child, we asked her to answer for the oldest child. The participants responded to the questionnaire items for themselves and their term-born children (mean age = 4.4, range = 3.0–6.2 years, *SD* = 0.8, 51 males and 56 females). No participant reported having a history of psychiatric or neurological conditions. Socioeconomic status (SES) was determined by family income and maternal education. Family income was rated on a 12-point scale, and maternal education was rated on a 5-point scale. We separately converted the two scores to z-scores and averaged them to create a total SES score (Moriguchi and Shinohara, 2019). The present study was conducted according to the guidelines provided in the Declaration of Helsinki, and written informed consent was obtained from the parents of each child before any assessment or data collection.

### Measures

#### Mother's and Child's Persistence

The child's and mother's persistence was measured using the short grit scale (Grit-S) (Duckworth and Quinn, 2009), which has eight items and consists of subscales, including perseverance of effort and consistency of interest. The sum of both was used as the score for the overall grit. We used the Japanese version of the Grit-S scale, whose factor structure, validity, and reliability have been confirmed, with a 5-point Likert-type scale ranging from 1 (not at all like me) to 5 (very much like me) (Nishikawa et al., 2015), with total scores of perseverance of effort ranging from 4 to 20 (higher score indicates greater perseverance of effort), total scores of consistency of interest ranging from 4 to 20 (higher score indicates greater consistency of interest), and total scores of overall grit ranging from 8 to 40 (higher score indicates greater overall grit).

#### Mother's Parenting Style

We used a parenting style questionnaire based on Baumrind's concept of parenting styles (i.e., responsiveness and control) (Baumrind, 1967; Robinson et al., 1995). The Japanese version of the questionnaire consists of 13 items that loaded responsiveness (eight items) and control (five items) factors and has been validated in a previous study (Nakamichi, 2013). A 4-point Likert-type scale ranging from 1 (never) to 4 (always) was provided for each item, with total scores of responsiveness ranging from 8 to 32 (higher score indicates stronger tendency of mother's responsiveness) and total scores of control ranging from 5 to 20 (higher score indicates stronger tendency of mother's control).

#### Child's Self-Control

The present study used the Japanese short version of two subscales (Yamagata et al., 2006), inhibitory control and attentional focusing, which were representative of effortful control, in the Children's Behavior Questionnaire (CBQ; Rothbart et al., 2001). Inhibitory control consisted of six items that measure the ability to inhibit inappropriate behavior, and attentional focusing consisted of six items that measure the tendency to maintain attention to the task. The sum of both is used as the score for the total effortful control. A 5-point Likert-type scale ranging from 1 (not true) to 5 (true) was provided for each item, with total scores of inhibitory control ranging from 6 to 30 (higher score indicates greater inhibitory control), total scores of attentional focusing ranging from 6 to 30 (higher score indicates greater attentional focusing), and total scores of total effortful control ranging from 12 to 60 (higher score indicates greater total effortful control).

#### Child's Social Development

Children's emotional and behavioral problems were assessed using the Japanese version of the Strengths and Difficulties Questionnaire (SDQ) (Matsuishi et al., 2008), which was derived from the original version (Goodman, 1997). The SDQ is a 25-item questionnaire that measures behavioral strengths and difficulties of children and assesses five subscales (hyperactivity, peer relationship problems, conduct problems, emotional problems, and prosocial behaviors), each of which consists of five items. The total difficulty score can be calculated by summing the scores of the four subscales that measure children's weaknesses. A 3-point Likert-type scale ranging from 0 (not true) to 2 (certainly true) was provided for each item, with total scores of total difficulty ranging from 0 to 40 (higher score indicates more behavioral problems) and total scores of prosocial behavior ranging from 0 to 10 (higher score indicates more prosocial behavior).

## Data Analysis

To examine factors associated with the persistence in young children, the relationships among all the variable (children's age, SES, and Grit-S [child and mother], parenting style, effortful control, and SDQ scores) were tested using Spearman's rank correlation analysis, a statistical method for describing correlations in ordinal scales. We then conducted hierarchical multiple linear regression analyses to investigate the independent contributions of mothers' grit (overall grit, perseverance of effort, and consistency of interest) and parenting style (responsiveness and control) to children's grit (overall grit, perseverance of effort, and consistency of interest), effortful control (total effortful control, inhibition control, and attention focusing), and prosocial behavior and total difficulty score.

## Results

### Correlation Analysis Among Variables

The results of the correlation analysis are presented in Table 1. We found significant moderate positive correlations between young children and mothers for each score of Grit-S (i.e., overall grit, perseverance of effort, and consistency of interest) (ρ[105] = 0.31, *p* = 0.001; ρ[105] = 0.43, *p* = 0.001; ρ[105] = 0.29, *p* = 0.003). Furthermore, mothers' responsiveness was significantly positively correlated with children's overall grit and perseverance of effort (ρ[105]= 0.23, *p* = 0.019; ρ[105] = 0.30, *p* = 0.002). Child grit was mostly significantly correlated with effortful control (see Table 1). Mothers' overall grit and perseverance of effort were positively correlated with total difference scores (ρ[105] = 0.22, *p* = 0.025; ρ[105] = 0.21, *p* = 0.034). Mothers' overall grit, perseverance of effort, and consistency of interest were negatively correlated with the total difficulty score (ρ[105] = −0.36, *p* < 0.001; ρ[105] = −0.24, *p* = 0.014; ρ[105] = −0.36, *p* < 0.001).

**Table 1:** Correlation among variables.

	**1**	**2**	**3**	**4**	**5**	**6**	**7**	**8**	**9**	**10**	**11**	**12**	**13**	**14**	**15**
Demografic information														
1. Age	–	0.03	−0.05	0.12	−0.12	−0.08	−0.01	−0.05	0.01	−0.12	0.13	0.11	0.11	−0.12	−0.04
2. SES		–	0.07	0.10	0.05	0.06	0.15	0.06	0.08	0.00	0.15	0.11	0.14	0.06	−0.09
Mother's grit														
3. Overall grit			–	0.76**	0.84**	0.33**	0.20*	0.31**	0.30**	0.24*	0.46**	0.37**	0.43**	0.22*	−0.36**
4. Perseverance of effort				–	0.33**	0.26**	0.12	0.32**	0.43**	0.09	0.39**	0.28**	0.39**	0.21*	−0.24*
5. Consistency of interest					–	0.27**	0.16^+^	0.20*	0.08	0.29**	0.34**	0.32**	0.28**	0.14	−0.36**
Mother's parenting style														
6. Responsiveness						–	0.35**	0.23*	0.30**	0.03	0.53**	0.49**	0.45**	0.32**	−0.38**
7. Control							–	0.05	0.19*	−0.13	0.24*	0.24*	0.18^+^	0.22*	−0.26**
Child's grit														
8. Overall grit								–	0.78**	0.70**	0.44**	0.28**	0.51**	0.09	−0.34**
9. Perseverance of effort									–	0.16	0.41**	0.27**	0.46**	0.20*	−0.35**
10. Consistency of interest										–	0.27**	0.16	0.33**	−0.05	−0.18^+^
Child's effortful control														
11. Total effortful control											–	0.88**	0.87**	0.31**	−0.49**
12. Inhibition control												–	0.55**	0.24*	−0.43**
13. Attention focusing													–	0.30**	−0.44**
Child's strength and difficulties														
14. Prosocial behavior														–	−0.31**
15. Total difficulties score															–

**p < 0.01; *p < 0.05; ^+^p < 0.10.

### Mother's Persistence and Parenting Style Are Associated With Child's Persistence

To analyze the independent contributions of mothers' persistence and parenting style to children's persistence, hierarchical multiple linear regression analyses with the stepwise method were performed (Table 2). The control variables to be entered into the models were the child's age and SES.

**Table 2:** Results of hierarchical multiple linear regression analyses predicting child's persistence from mother's persistence and parenting style.

**Valuable**	**β**	***t*-value**	***p*-value**	**95% Confidence interval**	
				**Low bound**	**Upper bound**
Child's overall grit: *R*^2^ = 0.18					
Age	−0.12	−1.32	0.190	−0.004	0.001
SES	0.02	0.22	0.826	−0.884	1.104
Mother's overall grit	0.42	4.67	<0.001	0.328	0.813
Child's perseverance of effort: *R*^2^ = 0.20					
Age	−0.06	−0.66	0.513	−0.002	0.001
SES	0.31	0.31	0.761	−0.574	0.783
Mother's perseverance of effort	4.95	4.95	<0.001	0.248	0.579
Child's consistency of interest: *R*^2^ = 0.15					
Age	−0.05	−0.50	0.621	−0.002	0.001
SES	0.05	0.51	0.609	−0.462	0.785
Mother's consistency of interests	0.34	3.61	<0.001	0.119	0.409
Mother's control	−0.22	−2.37	0.020	−0.572	−0.050

Mothers' overall grit was significantly associated with their children's overall grit (β = 0.42, *p* < 0.001). The models explained 18% (*R*^2^), *F*_(3, 106)_ = 7.70, *p* < 0.001. Mothers' perseverance of effort was significantly associated with their children's perseverance of effort (β = 0.44, *p* < 0.001). The models explained 20% (*R*^2^), *F*_(3, 106)_ = 8.39, *p* < 0.001. Mothers' consistency of interest and control were significantly associated with their children's consistency of interest (β = 0.34, *p* < 0.001; β = −0.22, *p* = 0.020). The models explained 20% (*R*^2^), *F*_(4, 106)_ = 4.33, *p* = 0.003.

### Mother's Persistence and Parenting Style Are Associated With Child's Self-Control, Prosocial Behavior, and Difficulties

To analyze the independent contributions of mothers' persistence and parenting style to children's self-control, prosocial behavior, and total difficulty score, we performed hierarchical multiple linear regression analyses using the stepwise method (Table 3). The children's age and SES were entered as control variables.

**Table 3:** Results of hierarchical multiple linear regression analyses predicting child's self-control, prosocial behavior, and difficulties from mother's persistence and parenting style.

**Valuable**	**β**	***t*-value**	***p*-value**	**95% Confidence interval**	
				**Low bound**	**Upper bound**
Child's total effortful control: *R*^2^ = 0.42					
Age	0.17	2.27	0.025	0.188	2.806
SES	0.11	1.41	0.163	−0.392	2.298
Mother's overall grit	0.34	4.26	<0.001	0.230	0.632
Mother's responsiveness	0.42	5.35	<0.001	0.639	1.392
Child's inhibition control: *R*^2^ = 0.35					
Age	0.19	2.32	0.022	0.136	1.736
SES	0.11	1.36	0.178	−0.574	1.365
Mother's consistency of interest	0.23	2.80	0.006	0.080	0.467
Mother's responsiveness	0.46	5.62	<0.001	0.413	0.862
Child's attention focusing: *R*^2^ = 0.32					
Age	0.15	1.78	0.078	−0.081	1.501
SES	0.09	1.11	0.272	−0.360	1.266
Mother's overall grit	0.37	4.33	<0.001	0.143	0.386
Mother's responsiveness	0.29	3.39	0.001	0.161	0.617
Prosocial behavior: *R*^2^ = 0.10					
Age	−0.09	−0.93	0.355	−0.002	0.001
SES	0.04	0.46	0.649	−0.356	0.570
Mother's responsiveness	0.29	3.06	0.003	0.067	0.316
Total difficulties score: *R*^2^ = 0.28					
Age	−0.02	−0.27	0.792	−0.003	0.002
SES	−0.09	−1.11	0.268	−1.578	0.443
Mother's consistency of interest	−0.30	−3.43	0.001	−0.659	−0.176
Mother's responsiveness	−0.34	−3.94	<0.001	−0.837	−0.276

Mothers' overall grit, responsiveness, and child's age were significantly related to children's total effortful control (β = 0.46, *p* < 0.001; β = 0.42, *p* < 0.001; β = 0.17, *p* = 0.025). The models explained 42% (*R*^2^), *F*_(4, 106)_ = 18.21, *p* < 0.001. Mothers' consistency of interest and responsiveness and children's age significantly predicted children's inhibition control (β = 0.23, *p* = 0.006; β = 0.46, *p* < 0.001; β = 0.19, *p* = 0.022). The models explained 35% (*R*^2^), *F*_(4, 106)_ = 13.97, *p* < 0.001. Mothers' overall grit and responsiveness significantly predicted children's attention focusing (β = 0.37, *p* < 0.001; β = 0.29, *p* = 0.001). The models explained 32% (*R*^2^), *F*_(4, 106)_ = 11.76, *p* < 0.001.

Mothers' responsiveness was significantly related to children's prosocial behavior scores (β = 0.20, *p* = 0.003). The models explained 10% (*R*^2^), *F*_(3, 106)_ = 3.82, *p* = 0.015. Mothers' responsiveness and consistency of interest significantly predicted children's total difficulty scores (β = −0.34, *p* < 0.001; β = −0.30, *p* = 0.001). The models explained 28% (*R*^2^), *F*_(4, 106)_ = 9.68, *p* < 0.001.

## Discussion

We first demonstrated that the degree of the mother's persistence (overall grit and perseverance of effort) was related to the degree of the child's persistence (overall grit and perseverance of effort). In addition, the mother's high consistency of interest (part of grit scale) and low control (part of parental style) were associated with the child's high consistency of interest. Second, the mother's persistence and responsiveness to the child were associated with the child's self-control, behavioral difficulties, and prosocial behaviors.

Our findings have revealed mothers' persistence is an important factor in children's persistence and possible implications for understanding the developmental mechanisms contributing to the persistence in children aged 3–6 years. According to the social learning theory, observational learning is crucial (Bandura, 1977, 1986). Persistent mothers showed persistent behavior not only in caring for their children but also in every aspect of their daily lives, and their children routinely observed this behavior from their parents. Therefore, through observational learning, it is possible for children to learn and develop persistent behavior from their mothers' behaviors.

Importantly, we found that not only the mother's high consistency of interest but also the mother's low control was associated with the child's persistence (high consistency of interest). Previous studies have shown that children and adolescents tend to internalize and externalize problems when their mothers are more controlling (Baumrind, 1991; for review, see Chorpita and Barlow, 1998). Mothers who have more control over parenting and have excessive interference in the behavior of their children may reduce their willingness to set goals and autonomy in their goal-achieving behavior. In the context of the present study, high parental control might prevent children from continuing their persistent act on their own initiative.

Interestingly, we found that mothers' persistence and responsiveness were associated with children's self-control. This result suggests that a persistent mother may be more responsive to her child. As Bernier et al. (2010) showed, the generous support of the mother was involved in the development of the child's executive function; in our study, children of mothers with high persistence might be more likely to receive support from their mothers and grow up in an environment where they could concentrate on their activities and increase their persistence. Furthermore, the correlation between persistence and self-control in young children is shown. This result is consistent with prior studies on children, adolescents, and adults (Duckworth et al., 2007; Oriol et al., 2017), exploring the relationship between persistence and self-control. To achieve long-term goals, we need to regulate desires and exercise self-control; therefore, it is inevitable that children's persistence and self-control are positively correlated. Persistence and self-control may share mechanisms (Duckworth and Gross, 2014). For example, in terms of neural mechanisms, persistence and self-control may have a common neural basis (i.e., prefrontal cortex) (Rothbart et al., 2007; Posner and Rothbart, 2009; Hosoda et al., 2020). To elucidate the cognitive basis of grit, further examination should employ neural measurements that allow us to directly ascertain this possibility.

Our results showed that the mother's consistency of interest and responsiveness could also be related to children's behavioral difficulties. This is the first report to demonstrate that mothers' persistence (consistency of interest), as well as parenting style (responsiveness), are associated with low behavioral difficulties in young children. Our findings are consistent with theories of parenting, suggesting that consistency in parenting behaviors leads to better adjustment in children (Bornstein, 2006). In terms of social learning theory, consistent behaviors of parents lead to predictability of behaviors, which makes it easier for children to learn. In such an environment, children can increase their successful experiences and improve their self-efficacy (Bandura, 1977, 1986). From an attachment perspective, consistent parental behaviors may form a stable attachment for children (De Wolff and van Ijzendoorn, 1997; Fuertes et al., 2006; Moss et al., 2011).

The present study has several limitations. First, because the metacognition was immature in our participants (3–6 years old) and they were unable to self-evaluate for persistence (Smortchkova and Shea, 2020), and the coronavirus pandemic has also been occurring, children's persistence was scaled by their mothers through online investigation, which had the potential to contain parents' biases. Future studies should examine the reliability of using questionnaires to assess children's persistence using behavioral data that objectively and quantitatively demonstrate children's persistence in addition to parental reports. Second, we investigated the associations between mothers' persistence and their children's persistence; however, we were not able to verify whether environmental care or genetic factors were more likely. In the future, it is necessary to consider genetic factors (Willems et al., 2019). Third, although the current study focused on the association with the mother as a factor in the persistence in children, the association with the father and other microsystems (school, teacher, and peer) may be possible (Padilla-Walker et al., 2012; O'Neal, 2018; Li et al., 2020). Fourthly, the current study utilized a cross-sectional design. Finally, whilst we used grit as a measure of persistence, “resourcefulness” and “frustration tolerance and courage” could also be considered concepts related to persistence (e.g., Rosenbaum and Ben-Ari, 1985; Rosenbaum, 1990; Wong, 1995). Examining these issues reveals the importance of parental persistence in the developmental factors of children's persistence. In addition, it can lead to a better understanding of the role of persistence in children's behavioral problems and their associated parental persistence.

The present findings provide among the first direct evidence that a high level of mother's persistence is essential for a young child's high persistence. Our results also suggest that mothers' persistence and responsiveness can also be associated with the level of self-control and behavioral difficulties of their young children. We propose that, ultimately, using mothers' persistence as an index of children's persistence may promote the development of a new strategy for education for developing children's persistence with perspectives on parent–child relationships and suggest the importance of educational interventions that enhance parents' persistence.

## Data Availability Statement

The raw data supporting the conclusions of this article will be made available by the authors, without undue reservation.

## Ethics Statement

The studies involving human participants were reviewed and approved by the institutional review board (University of Tokyo, 348-5). All procedures involving human subjects in this study were approved by the Ethics Committee of Musashino University (R2-001). The patients/participants provided their written informed consent to participate in this study. Written informed consent was obtained from the individual(s) for the publication of any potentially identifiable images or data included in this article.

## Author Contributions

CH conceptualized. MI, AS, KO, and CH contributed to the design of the work. MI took part in the data acquisition. MI, AS, KH, and CH contributed to the analysis and interpretation of data of the work. MI, AS, and CH contributed to the drafting of this paper. All authors approved the final version for submission and agreed to be accountable for all aspects of the work to ensure that all questions related to the accuracy or integrity of any part of the work are appropriately investigated and resolved.

## Funding

This study was supported by grants from CREST (JPMJCR18A3) and Souhatsu from Japan Science and Technology Agency (JST) for CH, and JSPS KAKENHI (19K14392) and Musashino University Research (2020) for MI, and JSPS KAKENHI (18K02461) for AS.

## Conflict of Interest

The authors declare that the research was conducted in the absence of any commercial or financial relationships that could be construed as a potential conflict of interest.

## Publisher's Note

All claims expressed in this article are solely those of the authors and do not necessarily represent those of their affiliated organizations, or those of the publisher, the editors and the reviewers. Any product that may be evaluated in this article, or claim that may be made by its manufacturer, is not guaranteed or endorsed by the publisher.
